# Exosomes derived from BMSCs enhance diabetic wound healing through circ-Snhg11 delivery

**DOI:** 10.1186/s13098-023-01210-x

**Published:** 2024-02-07

**Authors:** Tao Tang, Linyi Chen, Ming Zhang, Chuang Wang, Xiaolong Du, Shenglin Ye, Xiaoqiang Li, Hong Chen, Nan Hu

**Affiliations:** 1 Department of Vascular Surgery, The Affiliated Nanjing Drum Tower Hospital, Nanjing University Medical School, #321 Zhongshan Road, Nanjing, Jiangsu, 210008 China; 2 Department of Ophthalmology, The Fourth Affiliated Hospital of Nanjing Medical University, #298 Nan Pu Road, Nanjing, Jiangsu, 210008 China

**Keywords:** Exosomes, Diabetic wound healing, Bone mesenchymal stem cells, circ-Snhg11, Endothelial progenitor cells (EPCs)

## Abstract

**Background:**

Exosomes (Exos) generated from bone mesenchymal stem cells (BMSCs) are elucidated to enhance cutaneous wound healing in mice models of diabetes mellitus (DM). While underlying mechanisms remain unknown.

**Methods:**

Next-generation sequencing (NGS) was used to examine changes in circRNA expression levels following Exo treatment. Luciferase assays were used to determine the interactions between RNAs. Immunofluorescence staining was used to examine reactive oxygen species (ROS) in endothelial progenitor cells (EPCs) cultured in high glucose (HG) conditions. Therapeutic effects regarding Exos were also examined by immunofluorescence.

**Results:**

We found that Exo treatment enhanced cutaneous wound healing significantly. NGS indicated that circ-Snhg11 was involved in Exo-mediated tissue repairing. Downregulation of circ-Snhg11 decreased Exo-mediated therapy responses during wound healing in diabetic mouse. Our luciferase reporter data confirmed that SLC7A11 and miR-144-3p were circ-Snhg11 downstream targets. miR-144-3p overexpression or SLC7A11 knockdown altered the protective effects of circ-Snhg11 upon EPCs exposed to HG conditions. Upregulation of circ-Snhg11 incremented therapy effects of Exo treatment during wound healing in DM mice through enhanced angiogenesis along with a reduction in GPX4-mediated ferroptosis.

**Conclusions:**

circ-Snhg11 in BMSC-Exos enhanced SLC7A11/GPX4-mediated anti-ferroptosis signals via miR-144-3p sponging resulting in enhanced diabetic wound healing and improved angiopoiesis.

## Background

Diabetes mellitus (DM) is a chronic trait. Based on a recent WHO epidemiological survey, the onset of DM among adults worldwide is predicted to increase from 6.4% (2010) to 7.7% (2030), while the number of affected adults is expected to increase to 439 million [1, 2]. Hyperglycemia-induced vascular damaging could be a main reason resulting in severe symptoms [2, 3]. Thus, novel therapeutic approach developments regarding diabetic ulcers is critical.

Mesenchymal stem cells (MSCs) are recently illustrated to have essential roles in wound repair and tissue regeneration [4]. Former investigations have reported that diabetic ferroptosis is critical in triggering inflammation [5]. Excessive inflammatory factor expressions disrupt angiogenesis [6, 7]. Autologous application of stem cells is applied for regenerative plastic surgery [8], and found to prevent ultraviolet (UV) radiation effects as well as skin photoaging [9]. Previous studies have found that the stromal vascular fraction (SVF) and decellularized extracellular matrices (ECM) may act as enhanced therapeutic medicinal products [10]. MSCs, biomaterials and platelet-rich plasma are novel regenerative approaches that can be used in chronic skin wounds as well as soft tissue defects [11, 12]. More and more investigations confirmed that exosomes (Exos) of MSCs have indispensable roles to regulate angiogenesis-based wound healing [13, 14]. Exos are a paracrine component and main contributor to stem cell efficacy [15]. Exos are small (40–50 nm) membrane particles originating from the endosomal system, and have indispensable functions for intercellular communication via the miRNAs, circRNAs, mRNAs and protein deliveries to recipient cells [16]. Importantly, circRNAs are involved in the regulation of Exo-mediated processes [17]. Furthermore, circRNAs regulate gene expression, and may therefore serve as candidate diagnostic and therapy biomarkers [18].

Former investigations demonstrated that Exos from bone MSCs (BMSCs) accelerate wound healing [19]. Here, we found that circ-Snhg11 expression, which is lowered in DM, was increased post-Exo treatment. Mechanistically, circ-Snhg11 enhanced SLC7A11/GPX4 expression via miR-144-3p sponging, thereby mediating anti-ferroptosis signals and resulting in accelerated diabetic wound healing and the promotion of angiopoiesis. Our findings also revealed that increased circ-Snhg11 expression in Exos heightened their therapeutic efficacy in accelerating diabetic wound healing.

## Methods

### BMSC isolation, characterization and culture

Femurs and tibias were collected from BALB/c mouse with sterile conditions. We isolated bone marrow filtrate to centrifuge them at 225 × *g*. Our team resuspend cells in low-glucose (LG)-DMEM (HyClone, Logan). The cell suspension was mixed with mouse lymphocyte separation medium with ratio 1:1, which we centrifuged at 1000×*g*. Milky turbid mononuclear cell layers were extracted. Our group resuspended cells in LG-DMEM with no FBS, which we centrifuged at 225 × *g*. We then resuspended pelleted cells in LG-DMEM complete medium including FBS, which we cultured in atmosphere containing 5% CO_2_ and saturated humidity. We subcultured cells with ratio 1:3 and cell confluency of 80 ~ 90%. Phase III or IV BMSCs were used in subsequent experiments. First generation cells were the first cells to be isolated, while second generation cells were frozen and used to seed subsequent cell populations. For phenotypic analyses, phycoerythrin (PE)-conjugated antibodies against CD105, CD29, CD44, CD90 and vWF were utilized. Immunoglobulin G-matched isotypes functioned as controls for antibodies.

### Multilineage BMSC differentiation

BMSC multilineage differentiation capability was confirmed by culturing phase III mouse BMSCs. To induce differentiation into adipocytes, our team cultured BMSCs in adipogenic differentiation medium. After two weeks, BMSC differentiations into adipocytes was examined by staining cells with ALP or alizarin red.

### BMSC-Exo isolation

Upon achieving 80–90% confluency, the group rinsed BMSCs in PBS, which we cultivated in EGM-2MV media without FBS and supplemented with 1× serum replacement solution. Our group collected medium that centrifuged at 2000×*g* to erase apoptosis cells and cellular debris followed by 12,000×*g* centrifugation for 0.5 h at 4 °C. Our team quantified exo protein content applying Pierce BCA Protein Assay Kit (LMAI Bio, China). Our team maintained BMSC-Exos at -80 °C or utilized them immediately in subsequent experiments. Western blotting and NTA were applied to characterize extracted Exos.

### Strand-specific NGS library preparation

Samples were obtained from the skin tissue of DM mice with or without Exo treatment (which involved injection with exosomes for 48 h). Our team extracted total RNA applying TRIzol reagent. We removed ribosomal RNA from ~ 3 µg total RNA for Illumina sequencing. Next, we digested RNA that purified with 40 U RNase R at 37 °C and RNA-seq library was made utilizing KAPA Stranded RNA-Seq Library Prep kit. Interactions among RNAs were predicted using a web-based tool.

### Cell culture and transfections

EPCs were purchased from ScienCell (#GD-C0162219). Cells were cultured in DMEM (Hyclone) including 10% FBS in a humidified incubator. Our team constructed circ-Snhg11 and SLC7A11 overexpression vectors through inserting SLC7A11 or circ-Snhg11 cDNA into a pcDNA3.1 vector. Genepharma (Suzhou, China) synthesized SLC7A11 siRNA and miR-144-3p mimics. We carried out transfection using Lipofectamine 2000 (Thermo Fisher Scientific) and cultured cells in DMEM containing 30 mM glucose to simulate a HG environment.

### RT-PCR and RNA isolation

We extracted total RNA from samples utilizing TRIzol Reagent [24]. We carried out PCR utilizing the 2× Taq PCR Master following protocols. Our group quantified fold alternations applying 2^−△△Ct^ approach. Following PCR primers were used:

circ-Snhg11: forward 5’-GTTCTGTGATGGTTCCTC-3’, reverse 5’-CAGCAGCGGAGTCCACG-3’ SLC7A11: forward 5’-ATACGCTGAGTGTGGTTTGC-3’, reverse 5’-CTTCATCCACTTCCACAGCG-3’; GPX4: forward 5’-ATACGCTGAGTGTGGTTTGC-3’, reverse 5’-CTTCATCCACTTCCACAGCG-3’; and GAPDH forward 5’-GCAAGGATGCTGGCGTAATG-3’, reverse 5’-TACGCGTAGGGGTTTGACAC-3’.

### Dual-luciferase reporter assay

Our group amplified circ-Snhg11/SLC7A11 3’UTRs including predicted miR-144-3p binding sites, which we inserted into pMIR-REPORT luciferase miRNA expression reporter vector. EPCs were co-transfected through 0.1 µg luciferase reporter vectors. Similar procedures were carried out for miR-control. Our team calculated relative luciferase activity via normalizing the firefly luminescence to *Renilla* luminescence 2 days post transfection.

### ROS analyses

ROS generation in EPCs or scar skin was quantified using 2’,7’-dichlorofluorescin diacetate. Briefly, 1 × 10^6^ EPCs cells were cultured in 100 mm culture dishes, stained with DAPI, which were incubated 20 mM DCF-DA for 10 min. Next, we treated cells with normoxia or hypoxia, and ROS production was detected by measuring DCF-DA fluorescence intensity.

### Tubule formation assay

Our team examined in vitro neovascularization in serum-starved EPCs incubated at 37 °C. Briefly, we observed tubular structures produced in Matrigel under a phase-contrast microscope. The tube lengths in 10 fields that selected randomly were measured.

### Diabetic wound induction

Our group induced diabetes in BALB/c male mouse via single intraperitoneal injection of 60 mg/kg streptozotocin (STZ) dissolved in 0.1 M citrate buffer. 3 days post STZ administration, we confirmed diabetes through capturing fasting blood glucose levels. We regarded mice having fasting blood glucose levels > 250 mg/dL as diabetic. These mice were stored for 1 month to stabilize blood glucose levels prior to subsequent experiments. Wounds were induced by first sterilizing hair on dorsal leg with povidone iodine solution, then our team made 4 mm full-thickness excisional wound. Mice were chosen randomly to access either 200 µg BMSC-Exos subcutaneous injection in PBS or PBS alone at four sites around wound. Mice were humanely sacrificed post 15 days and skin samples were cultivated. Every treatment group consisted of 6 mice.

### Immunohistochemical data

Our team incubated paraffin-embedded tissue sections over the night with primary antibodies over GPX4 or CD31, then stained with 3,3-diaminobenzidine.

### Statistical analysis

Statistician represented data by mean ± standard deviation (SD). Our team performed statistical analyses through GraphPad Prism (La Jolla, CA, USA) to define significances between groups. P-values ≤ 0.05 were considered as statistics significance. Our team employed 2-tailed Student’s t-tests to compute significances between 2 groups, while two-way ANOVA with post hoc Bonferroni tests or one-way ANOVA with Tukey tests were employed to define significant differences among > = 3 groups.

## Results

### BMSC-Exos and BMSCs characterization

**Fig. 1:**
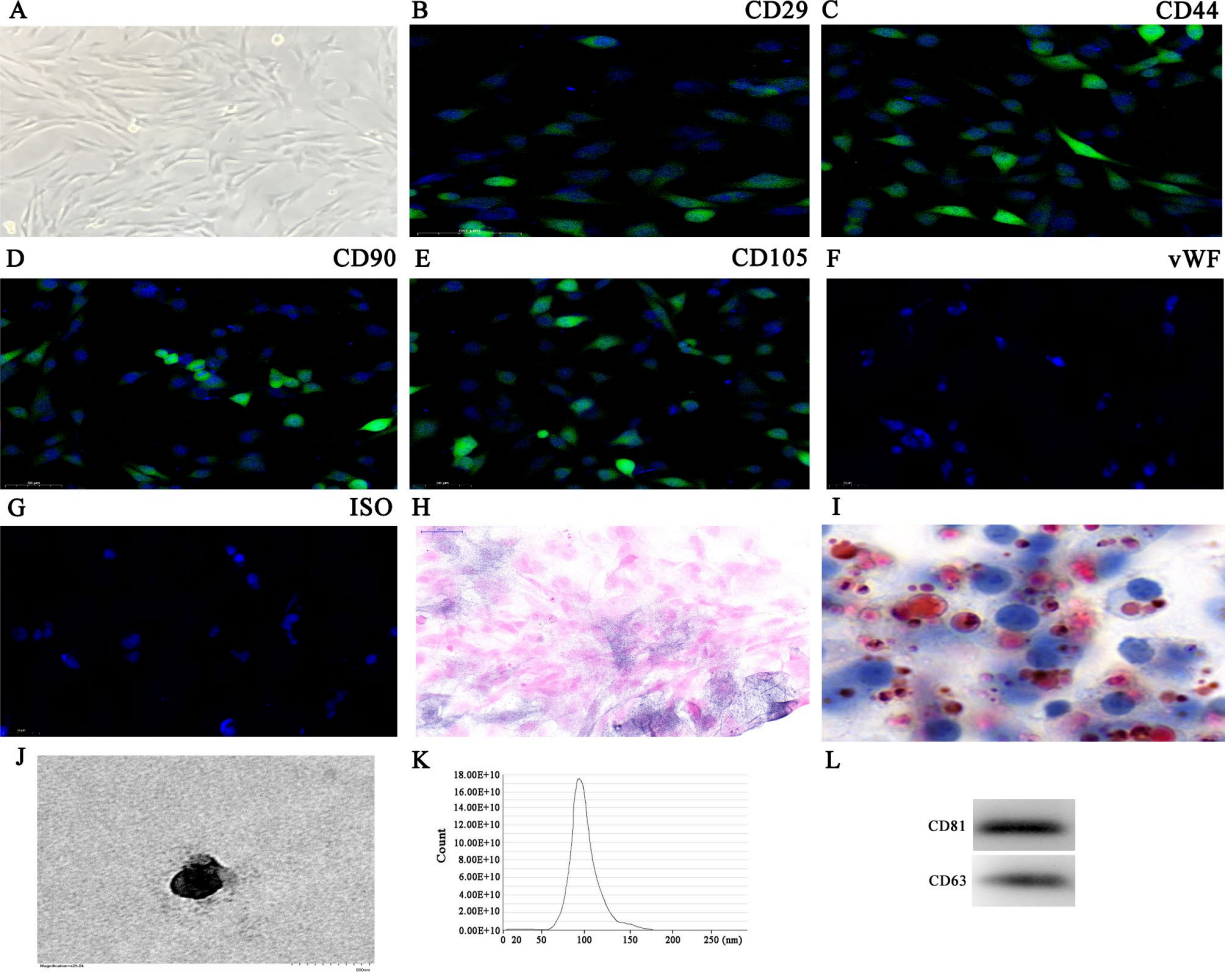
Characterization of Exos collected from BMSC recollection medium. (**A**) BMSCs displayed a classical cobblestone-like morphology. (**B-G**) Immunofluorescence staining for cell surface markers. Phycoerythrin-conjugated antibodies are shown in red (PE, red). CD90, CD105, CD44 and CD29 are positive, whereas vWF is negative. (**H-I**) The differentiation potential of BMSCs was assessed by alkaline phosphatase (**H**) and oil red O staining (**I**). (**J**) Transmission electron micrographs showing BMSC-Exo morphology. (**K**) The particle size distribution in purified BMSC-Exos was determined by NanoSight. (**L**) Representative western blots showing CD43 and CD81 expression levels in BMSC-Exos.

We extracted BMSCs with a typical fibroblast-like spindle shape (Fig. 1A). Immunofluorescence staining was positive for CD29 (#ab179471, 1:500, abcam, USA), CD105 (#ab221675, 1:500, abcam, USA), CD44 (#ab243894, 1:500, abcam, USA) and CD90 (#ab307736, 1:500, abcam, USA), but negative for the endothelial marker vWF (#ab287962, 1:500, abcam, USA) (Fig. 1B-G). Oil red O and ALP staining confirmed adipocyte and osteoblast differentiation potentials regarding BMSCs (Fig. 1H-I). BMSC-Exos showcased sphere- or cup-shaped morphology (Fig. 1J), consistent with previous studies [20]. NTA revealed that mean Exo diameter was ~ 100 nm (Fig. 1K). Western blot analyses were used to examine the Exo marker protein expressions, which included CD81 (#ab109201, 1:500, abcam, USA) and CD63 (#ab217345, 1:500, abcam, USA) (Fig. 1L).

### The effects of BMSC-Exo treatment on wound healing

**Fig. 2:**
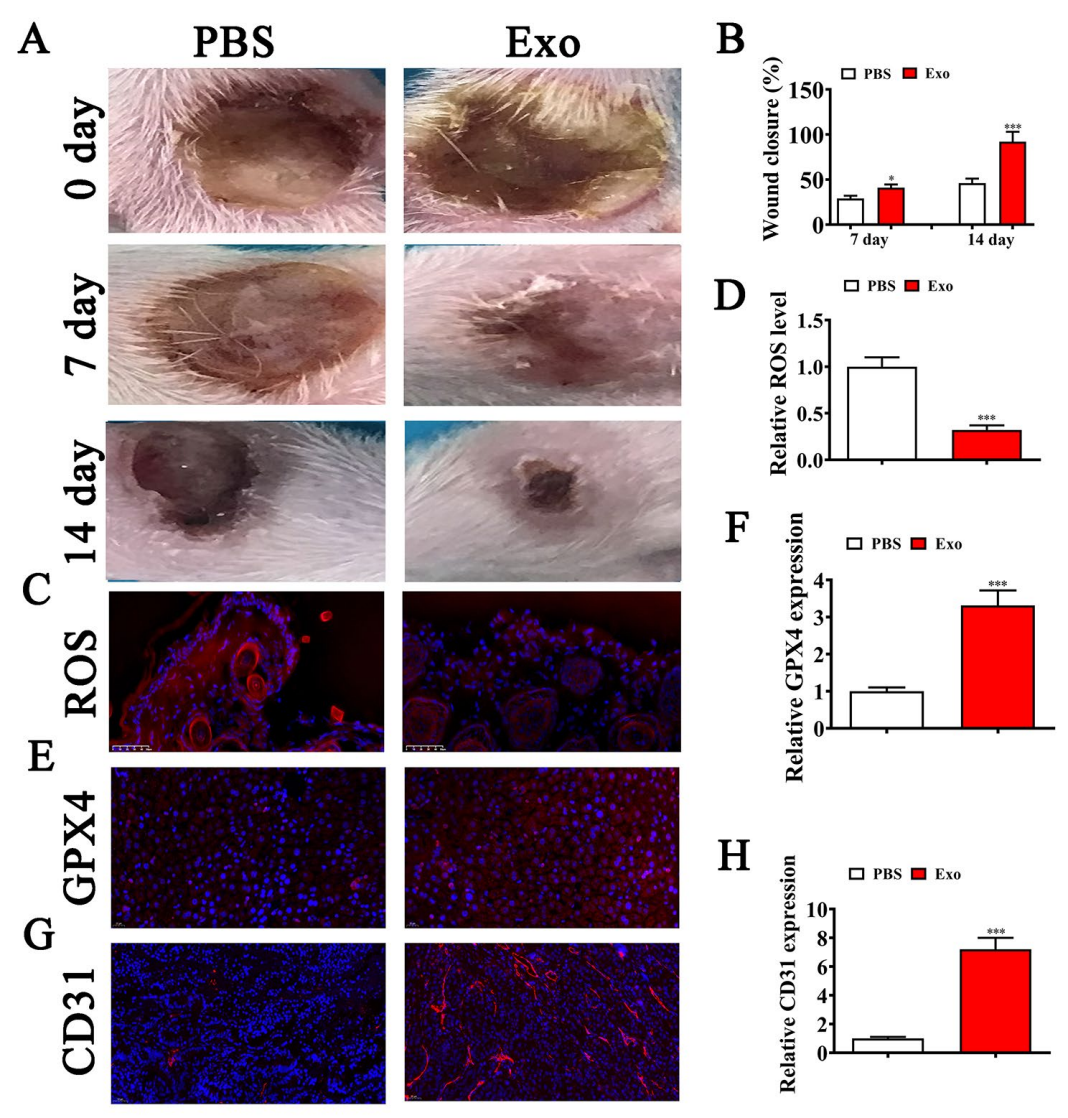
The effects of BMSC-Exo treatment on wound healing. (**A-B**) Representative images showing wound healing processes at distinct time points in different groups. Quantification of the wound healing rate. N = 6. (**C-D**) Immunofluorescence staining was used to assess ROS levels. Data are given as mean ± SD. (**E-F**) Immunofluorescence staining showing GPX4 expression levels in the ulcer site. (**G-H**) Immunofluorescence data showing CD31 expression. Data are presented as mean ± SD. ^*^p < 0.05, ^***^p < 0.001 vs. PBS group

BMSC-Exo treatment improved diabetic wound healing (Fig. 2A-B). Immunofluorescence analysis demonstrated that Exo treatment led to a decrease in ROS levels (Fig. 2C–D). In addition, GPX4 immunofluorescence staining revealed that Exo treatment resulted in a reduction in GPX4 expression (Fig. 2C–D), suggesting that Exo treatment reduced HG-induced ferroptosis. Our CD31 immunofluorescence data demonstrated that Exo treatment led to incremented CD31 expression at ulcer sites (Fig. 2E–F), suggesting that Exo treatment reversed HG-induced dysfunction.

### A role for circ-Snhg11 regarding BMSC-Exo-mediated wound repairing

**Fig. 3:**
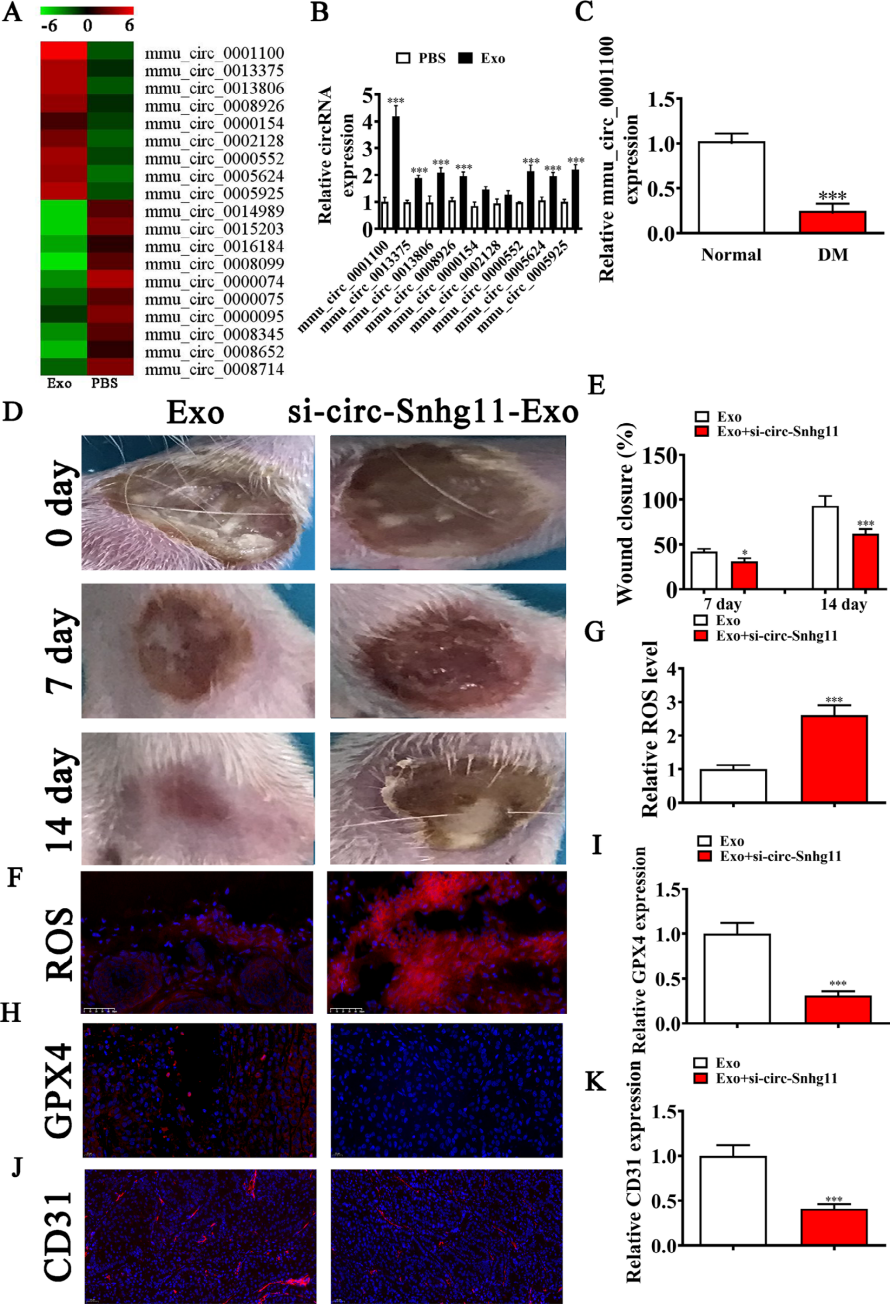
mmu_circ_0001100 (circ-Snhg11) has indispensable roles in BMSC-Exo-mediated wound repair. (**A**) NGS identified several downregulated and upregulated circRNAs in the ulcer site between the Exo and PBS treatment groups. (**B**) RT-qPCR showing upregulated circRNA expression levels in the ulcer site betwen the Exo and PBS treatment groups. (**C**) RT-qPCR data showing differences in mmu_circ_0001100 expression between normal and DM mouse serum. (**D-E**) Representative images showing wound healing processes at distinct time points in different groups. Quantification of the wound healing rate. N = 6. (**F-G**) Immunofluorescence data showing ROS levels in the ulcer site. (**H** and **I**) Immunofluorescence data showed GPX4 expression levels. (**J-K**) Immunofluorescence data showing CD31 expression in the ulcer site. Data are expressed as mean ± SD. ^*^p < 0.05, ^***^p < 0.001 vs. Exo group

NGS analysis revealed that Exo treatment led to circRNA expressions that not normal in diabetic mouse (Fig. 3A). Specifically, RT-qPCR analysis indicated that comparing to PBS alone group, Exo treatment led to high circ-Snhg11 expression (Fig. 3B). However, RT-qPCR revealed that circ-Snhg11 expression levels were reduced in the serum (Fig. 3C). We found that silencing circ-Snhg11 expression led to a reduction in the Exo-mediated protective effects, which accelerated diabetic wound healing (Fig. 3D–E). Furthermore, circ-Snhg11 knockdown restored ROS levels as measured by immunofluorescence analysis (Fig. 3F–G). In addition, GPX4 immunofluorescence staining revealed that circ-Snhg11 silencing reversed Exo treatment effects upon GPX4 expression levels (Fig. 3H, I). Similarly, CD31 circ-Snhg11 silencing reversed Exo treatment-induced CD31 expression (Fig. 3J, K). Together, these findings suggested that circ-Snhg11 has a critical role during BMSC Exo-mediated wound repairing.

### SLC7A11 is essential for BMSC Exo-mediated wound repair

**Fig. 4:**
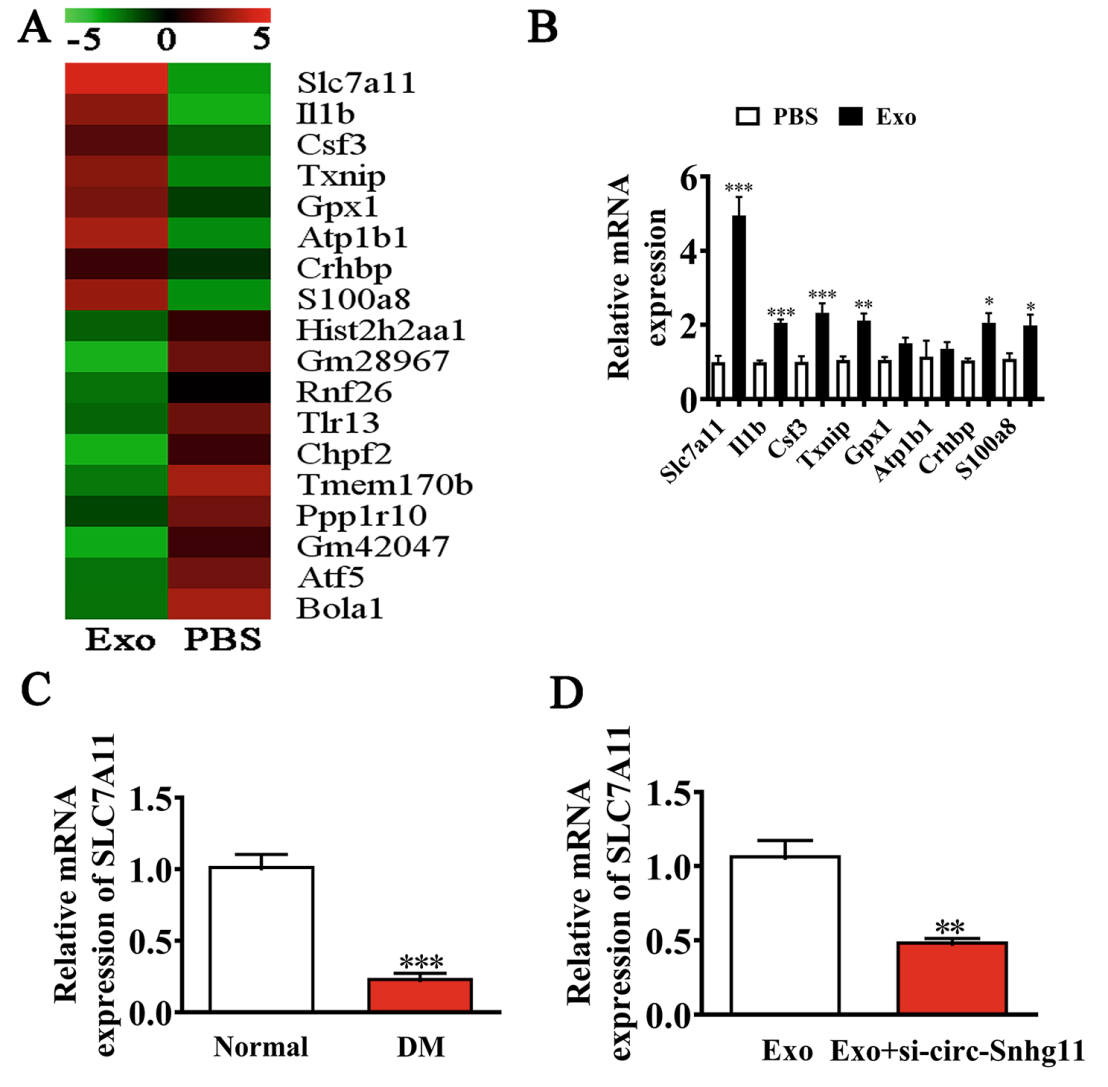
SLC7A11 has a key role in BMSC-Exo-mediated wound repair. (**A**) NGS identified several downregulated and upregulated mRNAs between the Exo and PBS treatment groups. (**B**) RT-qPCR showing upregulated mRNAs between the Exo and PBS treatment groups. (**C**) RT-qPCR comparing SLC7A11 expression levels between normal and DM mice serum. (**D**) RT-qPCR demonstrated SLC7A11 expression. Data are presented as mean ± SD. ^*^P < 0.05, ^**^P < 0.01, ^***^P < 0.001 vs. Exo group

NGS indicated that Exo treatment led to abnormal expression of mRNA in diabetic mouse (Fig. 4A). RT-qPCR revealed that comparing to PBS alone groups, Exo treatment increased SLC7A11 expression levels (Fig. 4B). RT-qPCR analysis further illustrated that SLC7A11 expression levels were reduced in DM serum (Fig. 4C). circ-Snhg11 knockdown resulted in reduced SLC7A11 expression levels resulting in accelerated diabetic wound healing (Fig. 4D).

### SLC7A11 is circ-Snhg11 downstream target

**Fig. 5:**
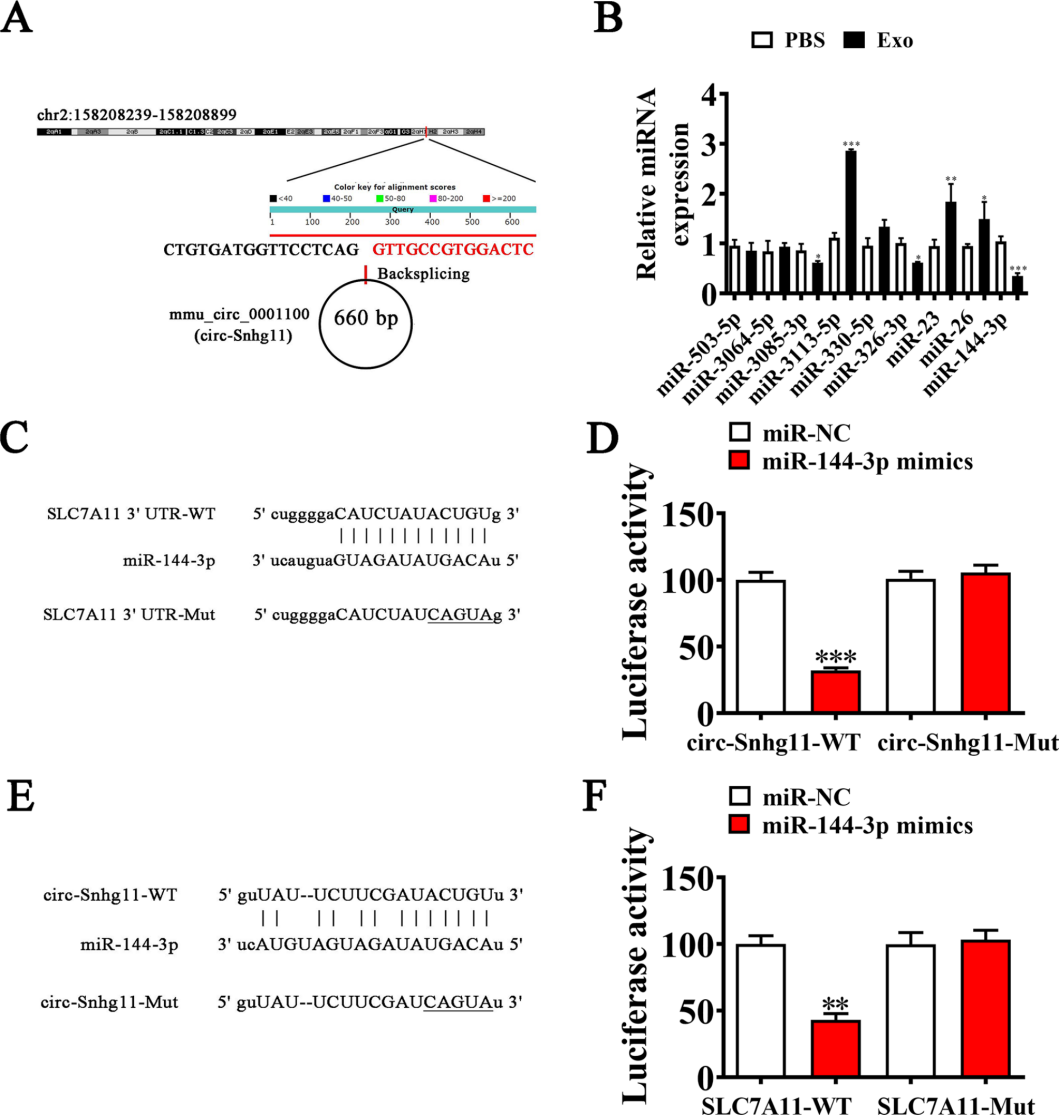
SLC7A11 and miR-144-3p are targets of circ-Snhg11. (**A**) Genomic loci showing the *Snhg11* gene and mmu_circ_0001100. (**B**) RT-qPCR showing the interactions between miRNAs. Data are given as mean ± SD. (**C**) Bioinformatics data predicting the miR-144-3p binding site on circ-Snhg11. MUT version of circ-Snhg11 is given. (**D**) Relative luciferase activity was determined 2 d post-HEK293T cell transfection with miR-144-3p mimic/NC or circ-Snhg11 WT/Mut. (**E**) miR-144-3p binding site predictions in SLC7A11 3’-UTR. MUT 3’-UTR-SLC7A11 is given. (**F**) Relative luciferase activity 2 d post-HEK293T cell transfection with miR-144-3p mimic/NC or 3’-UTR-SLC7A11 WT/Mut. Data are given as mean ± SD. ^*^P < 0.05, ^**^P < 0.01,^***^P < 0.001 vs. PBS

*In silico* data revealed that mmu_circ_0001100 is derived from *Snhg11* exons. Both *Snhg11* and spliced mature circRNAs are 660 bp. We denoted mmu_circ_0001100 as circ-Snhg11 (Fig. 5A). RT-qPCR analysis indicated that miRNAs interacted with the 3’UTR of SLC7A11 and circ-Snhg11. Specifically, a reduction in miR-144-3p expression levels was observed following Exo treatment (Fig. 5B).

Bioinformatics analysis indicated that circ-Snhg11 may function with miR-144-3p. Our luciferase reporter outcomes demonstrated that miR-144-3p inhibited luciferase activity in WT, but not MUT cells (Fig. 5C-D), suggesting that miR-144-3p may be a circ-Snhg11 target.

Bioinformatics analyses also revealed that SLC7A11 was a miR-144-3p downstream target. To further examine correlation between SLC7A11 and miR-144-3p, we inserted MUT or WT 3’UTR-SLC7A11 sequences into luciferase reporter vector (Fig. 5E), which we transfected into EPCs in presence or absence of miR-144-3p mimics. The team discovered that miR-144-3p suppressed luciferase function in WT cells (Fig. 5F). Thus, SLC7A11 may be a miR-144-3p target.

### SLC7A11 knockdown or mir-144-3p overexpression reverses the protective effects of circ-Snhg11 in EPCs

**Fig. 6:**
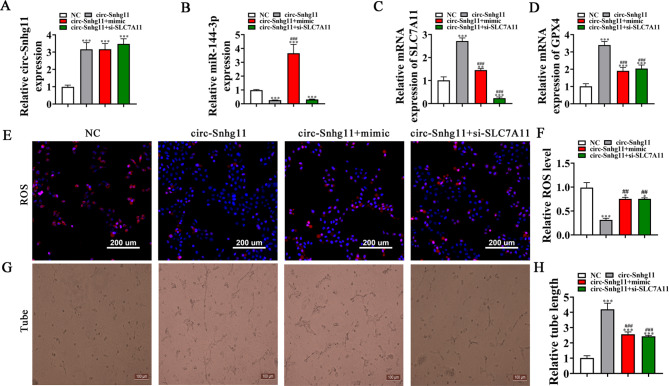
SLC7A11 downregulation or miR-144-3p overexpression reverses the protective effects of circ-Snhg11 on EPCs under high glucose conditions. (**A-C**) RT-qPCR showing circ-Snhg11 (**A**), miR-144-3p (**B**), and SLC7A11 (**C**) expression levels in EPCs. (**D**) RT-qPCR showing GPX4 expression in EPCs. (**E-F**) Immunofluorescence analysis assessing ROS levels in EPCs. (**G-H**) In vitro tube formation of EPCs. The total branching was analyzed. Data are given as mean ± SD. *P < 0.05, ***P < 0.001 vs. NC. ^###^P < 0.001 vs. circ-Snhg11

Our RT-qPCR data indicated that circ-Snhg11 expression levels were high following the circ-Snhg11 overexpression vector transfections. Nevertheless, treatment with miR-144-3p mimic or si-SLC7A11 had no effect on EPC circ-Snhg11 expression levels (Fig. 6A), telling that miR-144-3p and SLC7A11 are circ-Snhg11 downstream targets. SLC7A11 silencing had no effects on circ-Snhg11-induced miR-144-3p downregulation (Fig. 6B). Thus, miR-144-3p may be circ-Snhg11 downstream. In contrast, upregulation of miR-144-3p reversed circ-Snhg11 reduction effects post SLC7A11 expression. si-SLC7A11 treatment caused SLC7A11 expression level decrements (Fig. 6C). circ-Snhg11 may improve SLC7A11 expression via miR-144-3p sponging.

Our RT-qPCR data demonstrated that SLC7A11 downregulation or miR-144-3p overexpression (mimic) reversed the circ-Snhg11-induced increase in GPX4 expression in EPCs following HG condition exposures (Fig. 6D). Immunofluorescence data revealed that SLC7A11 downregulation or miR-144-3p overexpression (mimic) maintained ROS levels post-exposure to a HG microenvironment (Fig. 6E–F). SLC7A11 knockdown or miR-144-3p overexpression reversed circ-Snhg11-induced EPC angiogenic differentiation following HG condition exposures (Fig. 6G, H).

### SLC7A11 silencing or miR-144-3p overexpression reverses the effects of circ-Snhg11

**Fig. 7:**
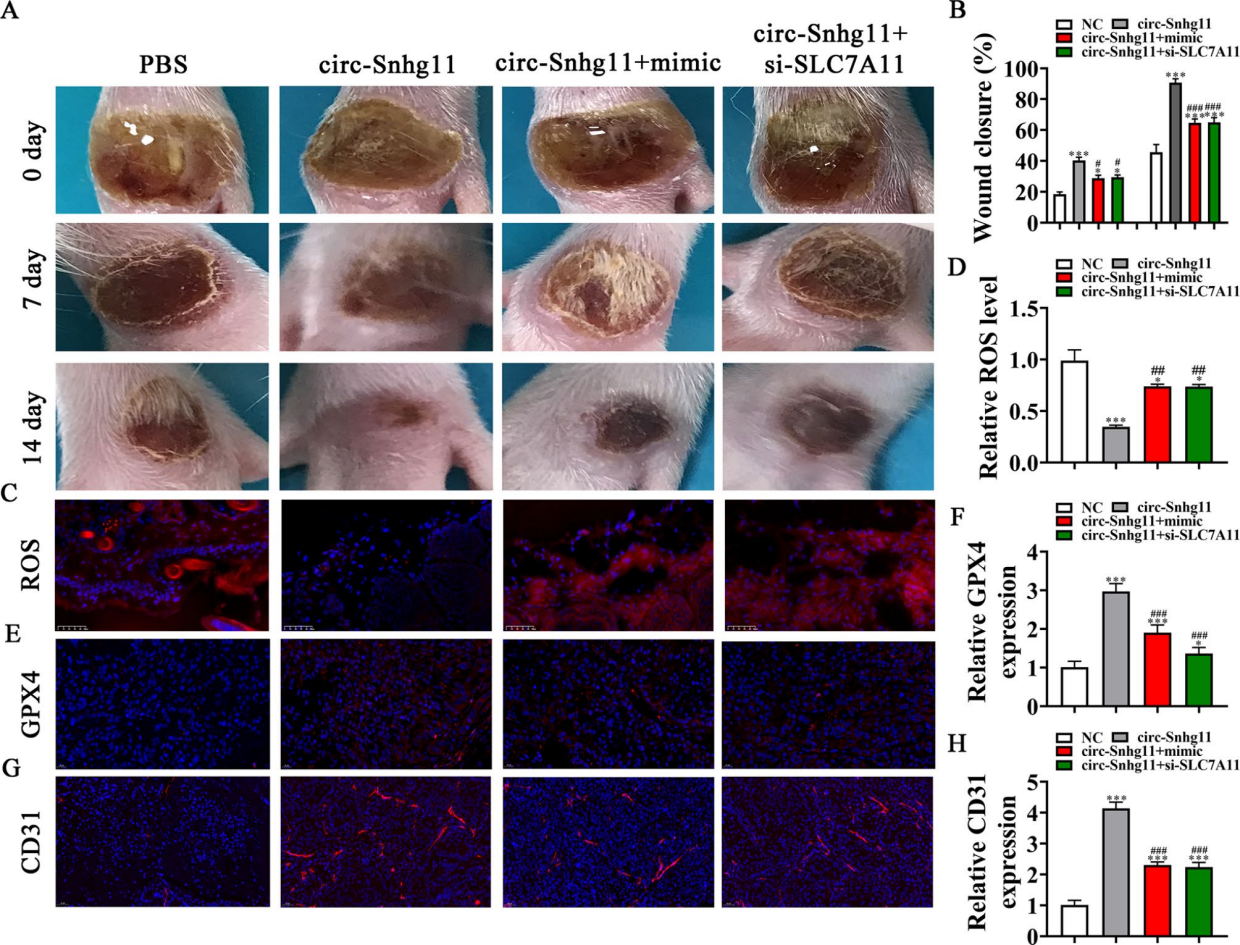
SLC7A11 downregulation or miR-144-3p overexpression reverses the protective effect of circ-Snhg11 mediated by Exo. (**A-B**) Representative images of wound healing processes at distinct time points in different treatment groups. Quantification of the wound healing rate. N = 6. (**C-D**) Immunofluorescence analysis was used to determine ROS levels. (**E-F**) Immunofluorescence data showing GPX4 expression. (**G-H**) Immunofluorescence data showing CD31 expression. Data are given as mean ± SD. ^*^p < 0.05, ^***^p < 0.001 vs. PBS group. ^#^P < 0.05, ^##^P < 0.01, ^###^P < 0.001 vs. circ-Snhg11

SLC7A11 silencing or miR-144-3p overexpression (mimic) reversed the Exo-circ-Snhg11 effects (Fig. 7A-B). Immunofluorescence staining revealed that SLC7A11 knockdown or miR-144-3p overexpression maintained ROS levels (Fig. 7C–D). GPX4 (Fig. 7E–F) and CD31 (Fig. 7G–H) immunofluorescence staining indicated that SLC7A11 silencing reversed the effects of Exo-circ-Snhg11 treatment.

**Fig. 8:**
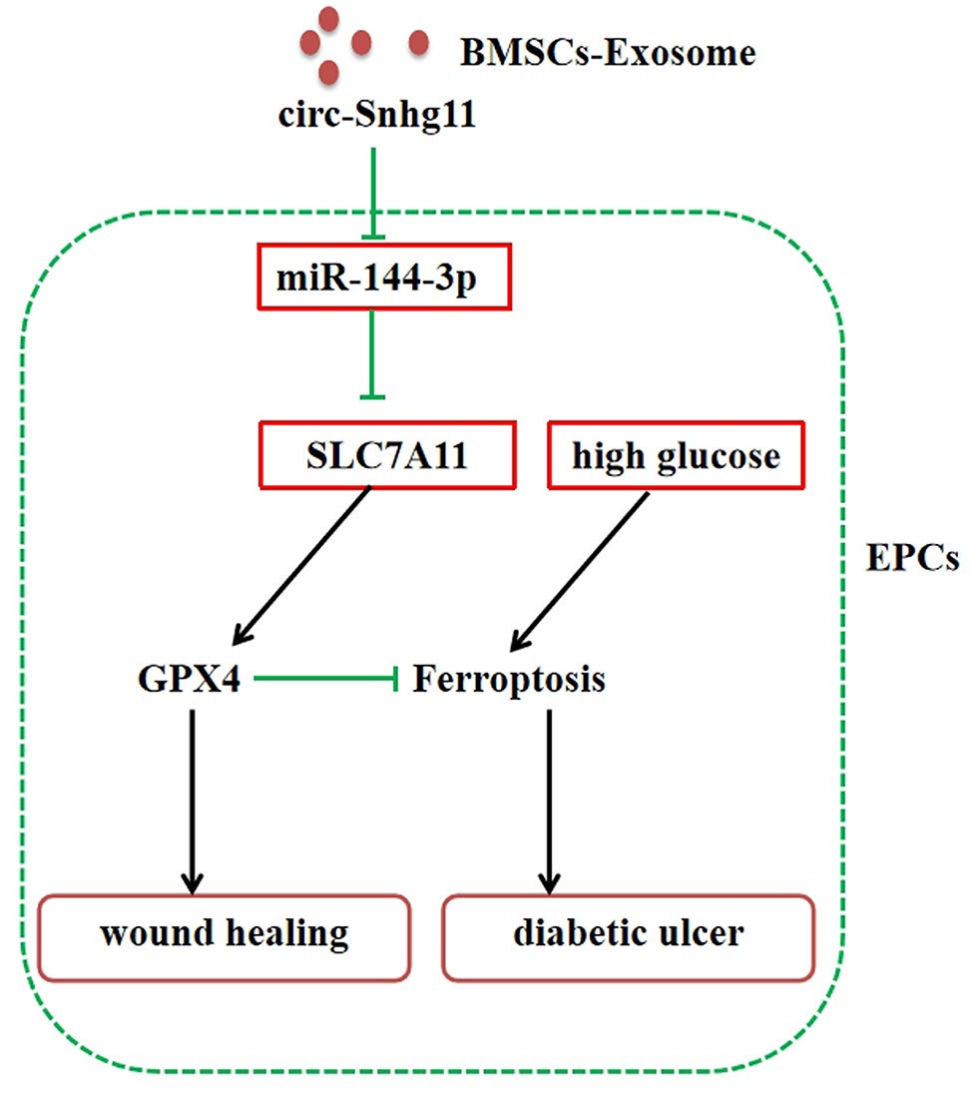
Graphic abstract for underlying mechanism

## Discussion

Accumulating evidence indicates that BMSCs possess candidate therapy effects that may improve wound healing [21]. BMSC-derived Exos are shown to exert their potential therapy effects during angiogenesis via their protein and miRNA cargos [22]. Previous studies have found that self-healing and antibacterial polypeptide-based FHE hydrogel can repair chronic wounds by multifunctional hydrogel with controlled Exo release [23]. Our findings suggest that pretreatment with BMSC-Exos improved diabetic wound healing. Specifically, Exo treatment enhanced angiogenesis and reduced ROS levels. In addition, we found that Exo treatment led to an increase in GPX4 expression.

Exosomes, extracellular vesicles with a diameter of 40 ~ 150 nm, can transport circRNAs, miRNAs, mRNAs, and growth factors [24]. More investigations described indispensable circRNA roles to regulate biological processes [25, 26]. Previous studies have confirmed that BMSC-Exos promoted the proliferation and migration of human corneal epithelial cells via activating the p44/42 MAPK pathway in vitro and also inhibited alkali burn-induced inflammation, fibrosis, and vascularization in corneal tissues in vivo [27]. The results showed that BMSC-derived exosomes or BMSC-exos promoted proliferation and migration and suppressed apoptosis in HaCaT cells damaged by H_2_O_2_ via the miR-93-3p/APAF1 axis [28]. Here, we found that Exo treatment enhanced circ-Snhg11 expression. Downregulation of circ-Snhg11 led to a decrease in Exo-mediated therapeutic effects through the suppression of angiogenesis and restoration of ROS levels. Sugestion that improving the stress microenvironment and promoting angiogenesis play an important role in promoting wound healing.

Our data further revealed that Exo treatment led to increased SLC7A11 expression. Previous studies have reported that SLC7A11 encodes the cystine/glutamate xCT transporter, and may therefore be a critical gene in the regulation of “iron overload-ferroptosis” [29]. Indeed, SLC7A11 was a ferroptosis inhibitor [30, 31]. Inhibition of ferroptosis can restore the function of vascular epithelial cells [32, 33]. Thus, our findings suggested that Exo treatment enhanced SLC7A11 expression, resulting in the inhibition of ferroptosis and restoration of EPC function, ultimately, promoting angiogenesis and wound healing.

The luciferase reporter outputs demonstrated that miR-144-3p interacted with SLC7A11. Previously, miR-144-3p expression was reported to suppress EPC mobilization [34]. Here, SLC7A11 downregulation or miR-144-3p overexpression reversed the circ-Snhg11-mediated protective effects upon EPCs, ultimately resulting in decreased angiogenesis and wound healing. Previously, mouse model studies have demonstrated that ferroptosis suppression enhanced inflammatory infiltration [5]. Hence, we can draw the conclusion that the better pro-angiogenic ability of BMSC-Exos was mediated by the circ-Snhg11/miR-144-3p/SLC7A11 pathway. However, there were several limitations to the present study. it remains to be determined how circ-Snhg11 expression in clinical patient. We also to identifield the effect of circ-Snhg11 to other cells beside EPCs.

## Conclusion

Our findings demonstrate that BMSC-derived Exos accumulate diabetic wound healing. Specifically, circ-Snhg11 expression enhances GPX4/SLC7A11-mediated anti-ferroptosis signals through sponging miR-144-3p, leading to accelerated diabetic wound repair and regeneration (Fig. 8). Our results offer a new perspective for the treatment of a diabetic wound as a cell-free therapy.

## Data Availability

The datasets analyzed are available upon requests to corresponding author.

## Declarations

### Statement

A preprint has been published [Tang et al. 2022] [35].

## Abbreviations

Exo: Exosomes
BMSCs: Bone mesenchymal stem cells
DM: Diabetes mellitus
NGS: Next-generation sequencing
EPCs: Endothelial progenitor cells
HG: High glucose
MSCs: Mesenchymal stem cells
UV: Ultraviolet
SVF: Stromal vascular fraction
ECM: Extracellular matrices
ATMP: Advanced therapy medicinal products
STZ: Streptozotocin
